# Clinical assessment of the therapeutic effect of low-level laser therapy on chronic recurrent aphthous stomatitis

**DOI:** 10.1080/13102818.2014.966526

**Published:** 2014-10-21

**Authors:** Hristina Lalabonova, Hristo Daskalov

**Affiliations:** ^a^ Department of Maxillofacial Surgery, Faculty of Dental Medicine, Medical University of Plovdiv, Plovdiv, Bulgaria; ^b^ Department of Oral Surgery, Faculty of Dental Medicine, Medical University of Plovdiv , Plovdiv, Bulgaria

**Keywords:** oral mucosa, aphthous stomatitis, therapy, laser, LLLT

## Abstract

The aim of this study was to clinically assess the therapeutic effect of low-level laser therapy (LLLT) on chronic recurrent aphthous stomatitis (RAS) using a protocol we developed especially for the purpose. The study included 180 patients: group 1 (the study group) – 90 patients who received LLLT using a laser operating in the red spectrum (658 nm; in a non-contact mode; power output *P* = 27 mW; frequency *f*_1_ = 5.8 Hz, *f*_2_ – continuous waveform; time *T* = 1.14 min; dosage of 2 J/cm^2^ once daily); group 2 (controls) – 90 patients who received pharmacotherapy (Granofurin and solcoseryl given twice daily). The indices we assessed were pain intensity, erythema dynamics and epithelization time. Pain was completely managed in 55.6% of group 1 patients one day after therapy began, while it took three days to alleviate pain for 11.1% of the patients in group 2. The erythema was managed entirely in 24.4% of group 1 patients after the first session, while it did not change in any of the group 2 patients. Pain intensity and erythema had similar dynamics for both groups. In 5 days, 75.6% of group 1 patients showed complete epithelization, while in group 2 the process was completed in only 37.8% of patients. As a whole, the results we obtained using LLLT to treat chronic RAS were better than those obtained in the group receiving pharmacotherapy. Pain and inflammation were very effectively managed with LLLT with the parameters we used and epithelization was considerably accelerated.

## Introduction

Chronic recurrent aphthous stomatitis (RAS) is a disorder characterized by small recurrent ulcerations in the oral mucosa. The main symptom of which patients complain is intense pain. The etiology of the condition is unknown, with stress being the main factor presumed responsible for causing it. The ulcers are clearly defined, small round lesions with a red erythematous halo. They appear predominantly on the movable oral mucosa, especially the inner side of the lips and cheeks, tongue and soft palate. They often appear in clusters of two or three lesions. The pain may be so extreme as to interfere with talking and eating.[1,2]

Different therapeutic modalities have been tested for managing the condition.[2,3] Convissar [4] used laser therapy, and Mikhaĭlova et al. [5] laser acupuncture.

The effects of low-level laser therapy (LLLT) on the trophics and regeneration of the tissues have been convincingly demonstrated experimentally.[6] The biostimulating effect of lasers used in the low-energy range (in the order of mW/sm) is manifested in acceleration of regeneration processes. Lasers using red light induce powerful analgesic and anti-inflammatory effects. Healing of the ulcerations is mainly achieved by stimulating epithelial growth and angiogenesis.[2,7] For example, Pinheiro et al. [8] use LLLT in the treatment of different disorders in the maxillofacial region.

The aim of this study was to clinically assess the therapeutic effect of LLLT on RAS using a protocol developed by us especially for the purpose.

## Subjects and methods

### Subjects

The study included 180 patients with chronic RAS whom we treated between 2007 and 2012. The distribution of patients by sex was: 17.2% male patients and 82.8% female patients. The mean age of patients was 43.01 ± 1.25 years. Informed consent was obtained from all patients, and the study was approved by the ethical committee at the Medical University of Plovdiv.

The patients were randomly divided into two groups at the beginning of the study.

- Group 1 (the study group) consisted of 90 patients who received LLLT in the red spectrum (λ = 658 nm) using a protocol we developed especially for the study.
- Group 2 (control group) comprised 90 patients receiving conventional pharmacotherapy.

Exclusion criteria for Group 1 were: (1) all forms of leukoplakia and (2) proliferative processes of the oral mucosa.

### Therapeutic protocol

The therapeutic protocol for group 1 was as follows: the laser therapy was conducted using a SIX Laser TS diode laser system with an irradiation wavelength of 658 nm. This wavelength was chosen on the basis of reports of positive evidence for its effect.[6,9] The aphthous area received laser non-contact irradiation at an oblique angle with a 3 mm cone-shaped diode laser tip. The irradiated area was 0.5 cm^2^. The area of irradiation included the aphthous lesion and the mucosa adjacent to it (0.5 to 1 cm in diameter). The characteristics of the laser irradiation were as follows: power output *P* = 27 mW; frequency *f*1 = 5.8 Hz, *f*2 – continuous waveform; time *T* = 1.14 min; dosage of 2 J/cm^2^. We conducted a session a day until symptoms abated.

The therapeutic protocol for group 2 patients included application of Granofurin and solcoseryl twice daily until symptoms disappeared.

### Clinical assessment

The effect of each specific treatment was assessed by gauging the changes in pain intensity, erythema and epithelization time. The assessment was made at 1, 2, 3 and 5 days.

Pain: A 10-point visual analogue scale was used to measure pain dynamics, 0 points were scored for no pain; 1 to 5 points, for mild pain and 6 to 10 points, for severe pain.

Erythema: The presence, reduction and absence of erythema were recorded.

Epithelization: The assessment included absence, beginning and completion of epithelization.

### Statistical analysis

The statistical analysis of the results was performed with SPSS v. 17 and MS Office Excel 2003.

## Results and discussion

There were no significant correlations between patients’ sex and age and the studied parameters for all patients and within each group we compared. The group a patient belonged to had a strong inverse correlation with the study variables after treatment began. The results from the statistical analysis are presented in Table 1.

**Table 1.** Statistical analysis of the results.

	Severe pain					Mild pain					No pain				
	Group 1		Group 2			Group 1		Group 2			Group 1		Group 2		
Groups	*n*	%	*n*	%	*P*	*n*	%	*n*	%	*P*	*n*	%	*n*	%	*P*
Before treatment	90	100	90	100	NS	0	0	0	0	NS	0	0	0	0	NS
Day 1	10	11.1	60	66.7	<0.01	30	33.3	30	33.3	NS	50	55.6	0	0	<0.01
Day 2	0	0	60	66.7	<0.01	9	10	30	33.3	<0.01	81	90	0	0	<0.01
Day 3	0	0	18	20.0	<0.01	0	0	62	68.9	<0.01	90	100	10	11.1	<0.01
Day 5	0	0	0	0	<0.01	0	0	50	55.6	<0.01	90	100	40	44.4	<0.01
	Erythema					Erythema decreases					No erythema				
	Group 1		Group 2			Group 1		Group 2			Group 1		Group 2		
Groups	*n*	%	*n*	%	*P*	*n*	%	*n*	%	*P*	*n*	%	*n*	%	*P*
Before treatment	90	100	90	100	NS	0	0	0	0	NS	0	0	0	0	NS
Day 1	0	0	90	100	<0.01	68	75.6	0	0	<0.01	22	24.4	0	0	<0.01
Day 2	0	0	90	100	<0.01	15	16.7	0	0	<0.01	75	83.3	0	0	<0.01
Day 3	0	0	40	44.4	<0.01	0	0	50	55.6	<0.01	90	100	0	0	<0.01
Day 5	0	0	10	11.1	<0.01	0	0	60	66.6	<0.01	0	0	20	22.2	<0.01
	No epithelization					Initial epithelization					Epithelization				
	Group 1		Group 2			Group 1		Group 2			Group 1		Group 2		
Groups	*n*	%	*n*	%	*P*	*n*	%	*n*	%	*P*	*n*	%	*n*	%	*P*
Day 1	90	100	90	100	NS	0	0	0	0	NS	0	0	0	0	NS
Day 2	21	23.3	90	100	<0.01	69	76.7	0	0	<0.01	0	0	0	0	NS
Day 3	0	0	60	66.7	<0.01	65	72.2	30	33.3	<0.01	25	27.8	0	0	<0.01
Day 5	0	0	0	0	<0.01	22	24.4	56	62.2	<0.01	68	75.6	34	37.8	<0.01

Note: NS: non-significant.

The study variables – pain intensity, erythema and epithelization of the ulcers – had the following dynamics.

### Pain

Prior to therapy all patients in both groups complained of severe pain. Therapy started simultaneously for both groups.

**Figure 1.**
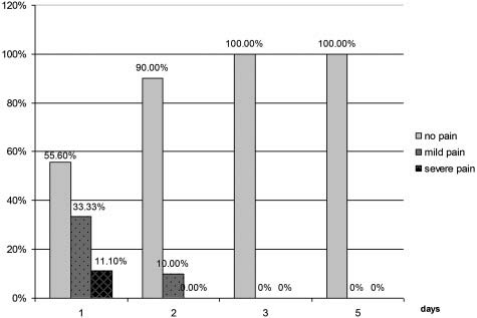
Results for the indicator ‘pain’ for group 1 (LLLT).

The dynamics of the indicator pain for patients in group 1 are shown in Figure 1. One day after initiation of the treatment, only 11.1% of group 1 patients experienced severe pain, 33.3% had mild pain and 55.6% felt no pain. On the second day, 90% of the patients were pain-free and 10% experienced mild pain. On the third and subsequent days all the group 1 patients were pain-free.

**Figure 2.**
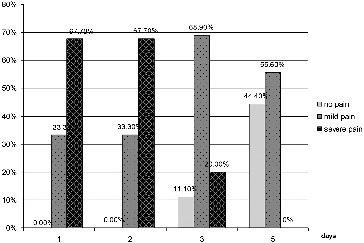
Results for the indicator ‘pain’ for group 2 (conventional pharmacotherapy).

The dynamics of the indicator pain for patients in group 2 (Figure 2) showed that one day after initiation of the treatment, none of the patients were free of pain, 67.7% of them experienced severe pain and 33.3% mild pain. On the second day, the results were the same. On the third day, only 11.1% were pain-free, 68.9% experienced mild pain and 20% had severe pain. On the fifth day, 44.4% were pain-free and 55.6% experienced mild pain.

### Erythema

Prior to therapy, all patients developed erythema around the aphthous lesion. At the beginning of treatment this parameter was identical for both groups.

**Figure 3.**
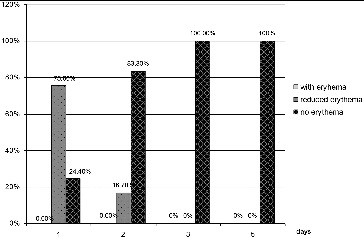
Results for the indicator ‘erythema’ for group 1 (LLLT).

The dynamics of the indicator erythema for patients in group 1 (Figure 3) demonstrated that one day after initiation of the treatment, 24.4% of the patients did not have erythema and 75.6% had reduction of the erythema. On the second day, 83.3% had no erythema, while 16.7% had reduction of the erythema. On the third and subsequent days none of the patients had erythema.

**Figure 4.**
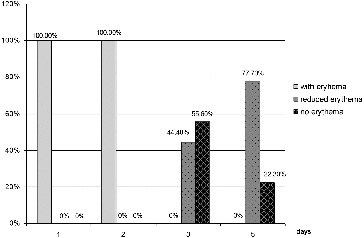
Results for the indicator ‘erythema’ for group 2 (conventional pharmacotherapy).

In contrast, the dynamics of indicator erythema for patients in group 2 (Figure 4) showed no sign of change in the erythema on the first and second day. On the third day, there was reduction of the erythema in 55.6% of the group 2 patients, while no changes were observed in the other 44.4%. On the fifth day, 22.3% of patients had no erythema and 66.6% had reduction of the erythema, whereas, there appeared to be no change in 11.1%.

### Epithelization

**Figure 5.**
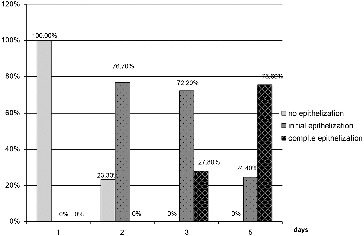
Results for the indicator ‘epithelization’ for group 1 (LLLT).

The dynamics of the indicator epithelization for patients in group 1 (Figure 5) did not reveal any changes on the first day. On the second day, initial epithelialization was visible in 76.7% of the patients. On the third day, there was initial epithelialization in 72.2% and in 27.8% the process was completed. On the fifth day, only 24.4% of the group 1 patients were still at the stage of initial epithelialization, whereas in 75.6% the epithelisation process was completed.

**Figure 6.**
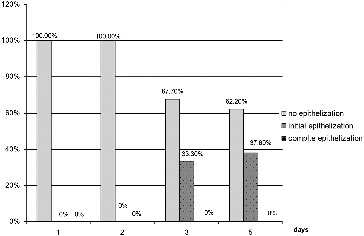
Results for the indicator ‘epithelization’ for group 2 (conventional pharmacotherapy).

In group 2, the dynamics of the indicator epithelization (Figure 6) did not show signs of improvement on the first and second days. On the third day, initial epithelialization was observed in 33.3% of the patients, while the rest were without change. On the fifth day, there was initial epithelialization in 62.2% and in 37.8% it was completed.

### Final remarks

Pain, as a major symptom of the disease seriously, affects eating and speech resulting in deterioration of the quality of life. In group 1, pain was managed completely in 55.6% of the patients as early as one day after the beginning of therapy, the patients with no effect of the treatment being only 11.1%. In group 2 there was no patient with completely managed pain, while the patients for whom the conventional therapy gave no effect were 67.7%. At day 3 in group 1, 100% of the patients were pain-free, while the group 2 patients still felt some pain even at day 5 (Figures 1 and 2).

Erythema developing around the aphthous lesion is a sign of inflammation. The reduction of the size of erythema is a sign of a successful healing process. The RAS in 24.4% of the patients in group 1 was managed successfully after the first procedure, with the remaining cases showing different degrees of successful management of the condition; while in group 2 there were no patients in whom RAS was effectively managed. All group 1 patients at day 3 had their ulcers successfully treated, whereas no patient in group 2 had the condition managed. These results are similar to the ones we obtained in the assessment of pain. The two parameters changed in parallel in both groups.

Epithelization is a sign of healing. Epithelization in group 1 was completed five days after beginning of therapy in 75.6% of patients, while in group 2 it was completed in only 37.8% of them.

Thus, the results demonstrated indirectly that the disturbed eating and speech in group 1 patients were restored by day 3, while in group 2, by the end of the study (day 5), we observed complete re-epithelization in only a small part of the patients. As a whole, we found LLLT to give better results in the treatment of chronic RAS than conventional pharmacotherapy. The chosen wavelength (658 nm) and LLLT parameters led to very efficient management of pain and inflammation, and to considerable acceleration of the epithelization process. LLLT was shown to exert analgesic, anti-inflammatory and regenerative effects in managing chronic RAS, which is in good agreement with recent developments in LLLT for RAS treatment. Low-level laser can decrease the healing time, pain intensity and also decrease the time of pain relief in patients with aphtae.[10,11] LLLT also reduced the pain and the inconvenience of eating, drinking and brushing teeth for patients with RAS.[12] These promising results suggest that low-energy laser therapy applied with our technique can be considered a reliable therapeutic modality to treat chronic RAS.

## Conclusions

The technique we used in managing chronic RAS with LLLT showed greater efficacy than pharmacotherapy. Patients already felt better on the day following the first procedure. The light wavelength we used (658 nm) and the chosen parameters of the laser operation led to very efficient management of the pain and inflammation symptoms. In addition, epithelization was considerably accelerated. The obtained results indicate that low-energy laser therapy applied with this technique is a reliable therapeutic modality to treat chronic (RAS).
